# Supplementary material for: Natural Cross-Kingdom Spread of Apple Scar Skin Viroid from Apple Trees to Fungi
Source: Cells. 2022 Nov 20;11(22):3686. doi: 10.3390/cells11223686 (PMC9688150; doi:10.3390/cells11223686)
Supplement: Supplementary file 1 [file cells-11-03686-s001.zip › cells-2013520-supplementary.pdf]

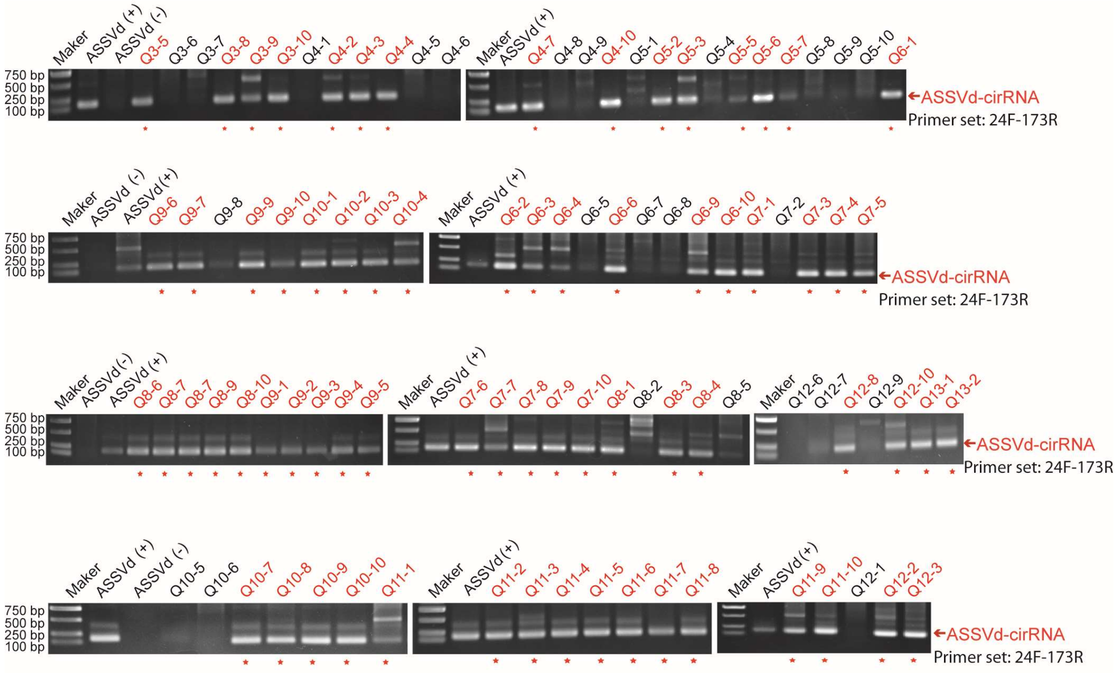

Supplementary Figure S1. Detection of ASSVd in fungal isolates by RT-PCR. A DNA ladder (Marker) was used as the size standard. ASSVd (−) and ASSVd (+) indicate the negative and positive control for the RT-PCR reaction, respectively, using RNA templates extracted from ASSVd-free and ASSVd-containing samples. RT-PCR primer sets are specific for ASSVd (Supplementary Table S1).

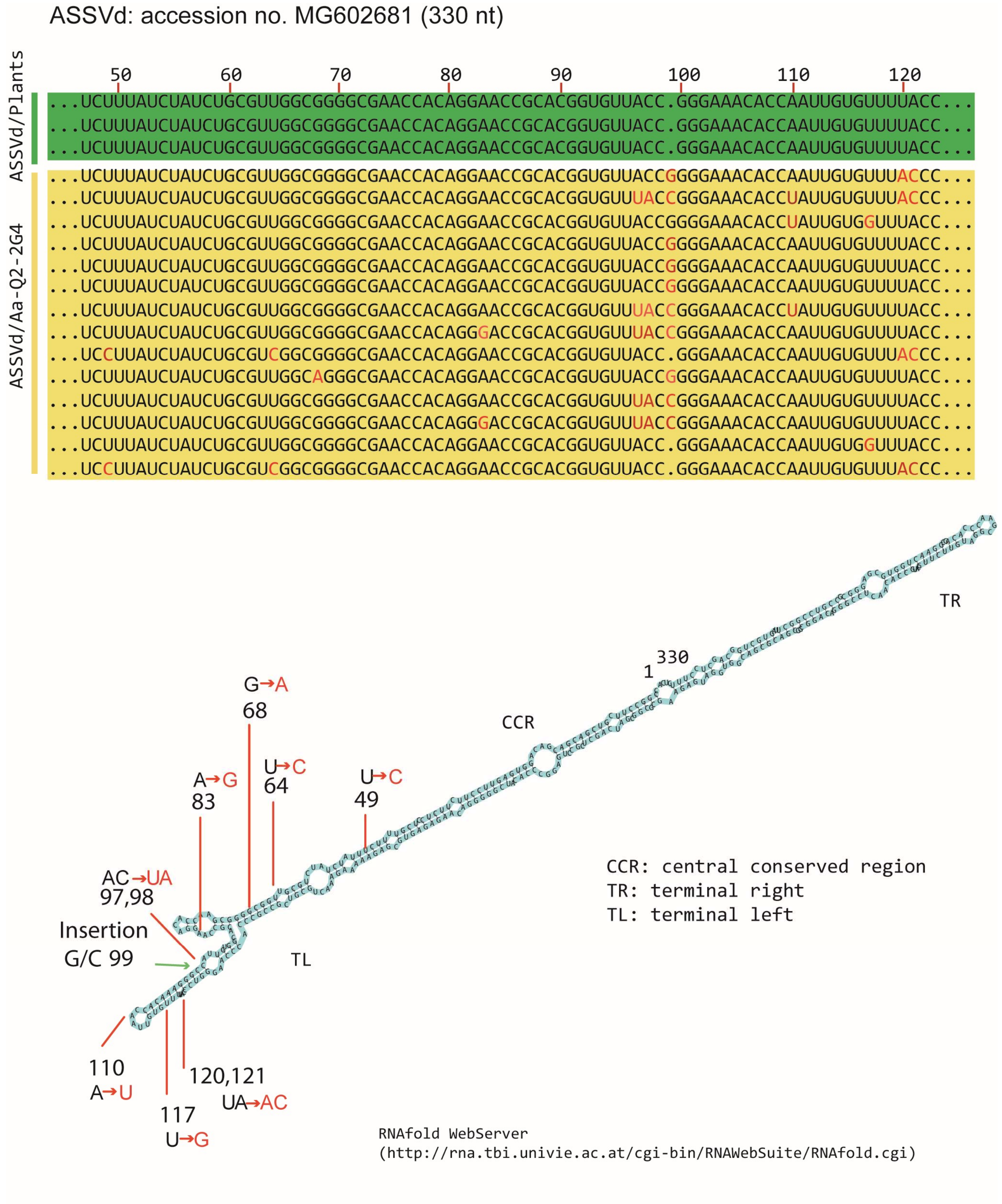

Supplementary Figure S2. Alignment of partial ASSVd genome sequences of the viroid strains detected in the plant and fungal isolates. The positions of the nucleotide substitutions in the ASSVd genome are illustrated below the alignment panel (Secondary structure prediction of ASSVd genome RNA).

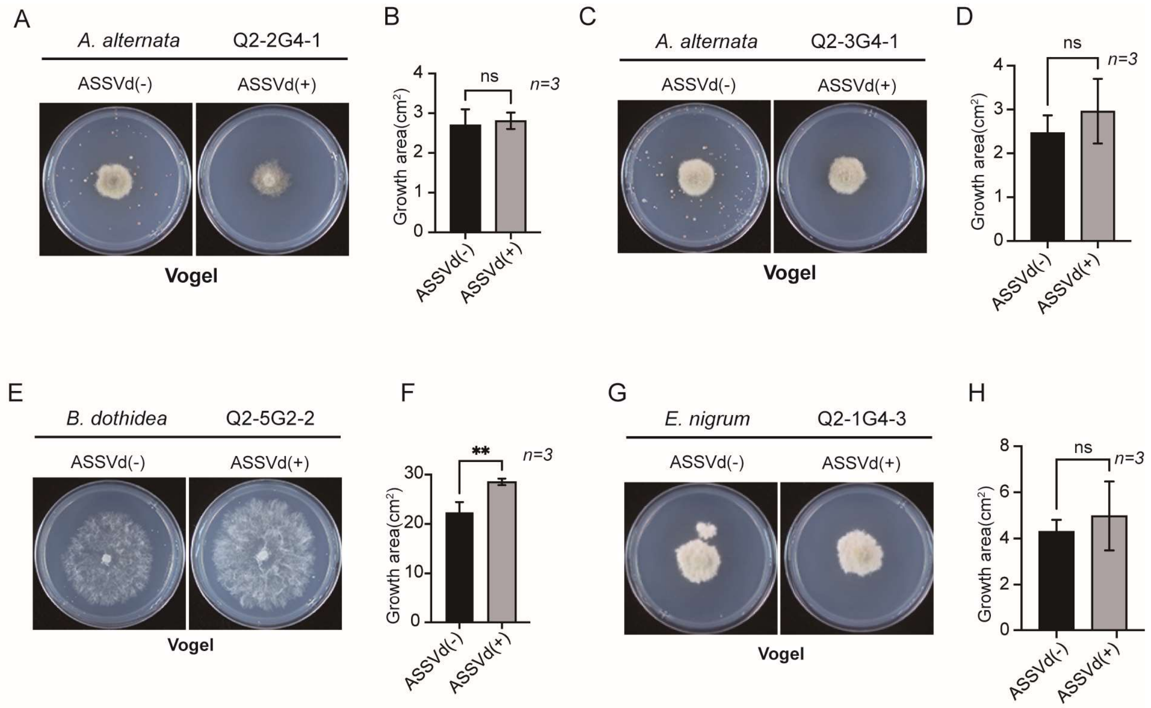

Supplementary Figure S3. The effects of ASSVd infection on fungal isolates grown on Vogel's minimal medium.

A, **C**, **E,** and **G.** Phenotypic growth of ASSVd-carrying and ASSVd-free fungal isolates. All isolates were grown on Vogel's minimal medium (6-cm plate) for 5–6 days and photographed. ASSVd (+) and ASSVd (−) indicate carrying and free isolates respectively. **B**, **D**, **F,** and **H.** The lesion area measured on inoculated apple leaves described in the data are presented as mean ± SD (n = 3). ** indicates a significant difference at $p < 0.01$ (Student's t-test).

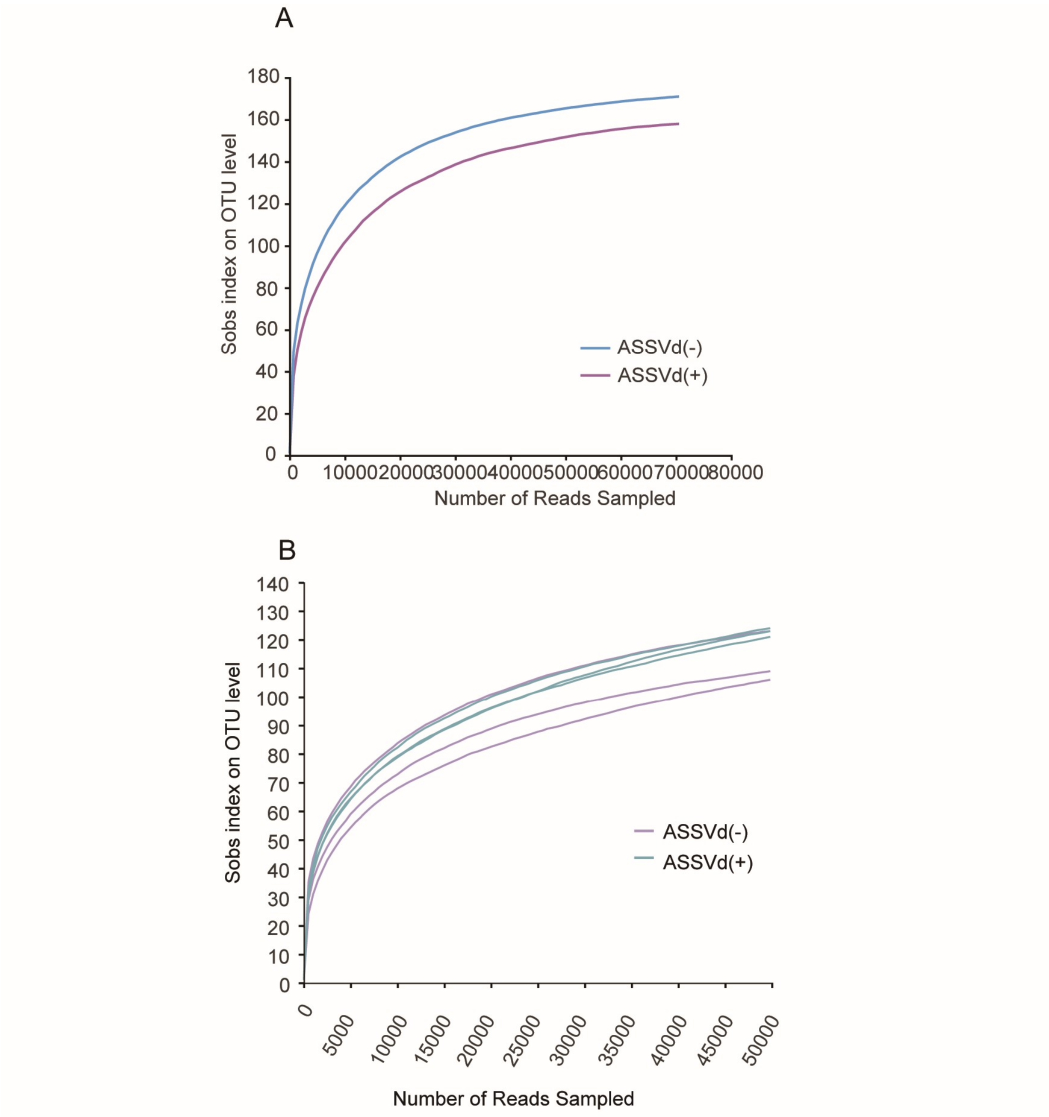

Supplementary Figure S4. The dilution curves based on the Sobs index at the OTU **level.**

A. ASSVd-infected (+) and ASSVd-free (−) samples collected in the autumn of 2019.

B. ASSVd-infected (+) and ASSVd-free (−) samples collected in the spring of 2020.

Supplementary Table S1. List of primers used in this study.

Primer Name	Oligonucleotide sequence (5’- 3’)	Size of PCR product
F-MdEF-1α	ATTCAAGTATGCCTGGGTGC	174 bp
R-MdEF-1α	CAGTCAGCCTGTGATGTTCC	
ASSVd-1F	CCGGCCTTCGTCGACGACGA	330 bp
ASSVd-330R	TGAGAAAGGAGCTGCCAGCAC	
ASSVd-173R	TGTTCTCTCACGCTCTTTTTCTTT	150 bp
ASSVd-24F	GTGAGTTCCTTCTTCTCCTCGTTTT	
ITS4R	TCCTCCGCTTATTGATATGC	608 bp
ITS1F<br>ITS2R	CTTGGTCATTTAGAGGAAGTAA<br>GCTGCGTTCTTCATCGATGC	282 bp

Supplementary Table S2. List of software and databases used for the data analysis in this study.

Analysis software / Database	Version number	Function	Link
Flash	1.2.11	Splicing paired-end sequences	https://ccb.jhu.edu/software/FLASH/index.shtml
Qiime	1.9.1	Generating tables of abundances at each taxonomic level; calculating the distances of beta diversity	http://qiime.org/install/index.html
Uparse	7.0.1090	OTU clustering	http://www.drive5.com/uparse/
RDP Classifier	2.11	For sequence classification annotation	https://sourceforge.net/projects/rdp-classifier/
Usearch	7	OTU statistics	http://www.drive5.com/usearch/
Mothur	1.30.2	Alpha diversity analysis	https://www.mothur.org/wiki/Download_mothur
UNITE	8	ITS database of fungal	https://unite.ut.ee/
FunGene	9.6	Functional gene databases	http://www.fungene-db.fr/
Funguild	1	Annotation databases of fungal functional	http://www.funguild.org/
MAFFT	7.2	Multiple sequence alignment	https://mafft.cbrc.jp/alignment/software/
Fastp	0.19.6	Quality control	https://github.com/OpenGene/fastp

Information of the analysis software / database for each analysis in this paper.

Supplementary Table S3. Summary of fungal amplicon sequencing data.

Collecting time	Places / plant number	Name	Raw reads	Trimmed Sequence reads	OTUs	Genus
Harvesting stage (2019)	Site 1-3 /9	ASSVd(-)	146892	73446	171	75
	Site 1-3/9	ASSVd(+)	143268	71634	158	74
Flowering stage (2020)	Site 1/3	ASSVd(-)	126006	63003	123	56
	Site 1/3	ASSVd(+)	100488	50244	125	54
	Site 2/3	ASSVd(-)	142772	71386	106	47
	Site 2/3	ASSVd(+)	123812	61906	123	48
	Site 3/3	ASSVd(-)	122600	61300	109	49
	Site 3/3	ASSVd(+)	141552	70776	121	51

Supplementary Table S4. Relative abundance of the 10 most abundant fungal groups (genera) present in ASSVd-infected and ASSVd-free apple stem samples collected in the autumn of 2019.

**Fungal Genus**	**ASSVd(-) %**	**ASSVd(+) %**
Genera in Phaeosphaeriaceae	32.69	8.26
Cladosporium	15.43	22.62
Setomelanomma	7.84	17.80
Pyrenochaeta	1.01	22.64
Alternaria	6.30	16.53
Erythrobasidium	10.18	1.03
Leotionmycetes	6.97	0.37
Didymellaceae	1.66	2.39
Neosetophoma	3.34	0.43
Filobasidium	0.82	1.93
Others	13.76	6.00

Supplementary Table S5. Relative abundance of the 10 most abundant fungal groups (genera) present in ASSVd-infected and ASSVd-free apple stem samples collected in the spring of 2020.

**Fungal Genus**	**Site 1**		**Site 2**		**Site 3**	
	**ASSVd(-) %**	**ASSVd(+) %**	**ASSVd(-) %**	**ASSVd(+) %**	**ASSVd(-) %**	**ASSVd(+) %**
Genera in Phaeo-sphaeriaceae	17.39	44.68	64.76	61.59	49.39	50.7
Alternaria	**12.85**	**22.67**	**3.02**	**11.65**	**6.67**	**11.88**
Genera in Pleosporales	24.64	9.42	12.23	2.3	0.31	6.73
Cladosporium	13.56	7.95	1.52	10.58	3.14	4.538
Genera in Didymel-laceae	4.13	5.55	11.16	0.61	8.87	10.76
Setophoma	1.64	0.62	0.04	2.34	12.36	1.04
Erythrobasidium	**4.03**	**2.53**	**2.68**	**1.98**	**1.92**	**1.03**
Chaetosphaeronema	**5.31**	**0.41**	**0.39**	**0.21**	**5.80**	**0.06**
Vishniacozyma	0.52	0.46	0.25	1.05	7.06	0.95
Pyrenochaeta	0.18	0.49	0.24	0.02	0.04	6.67
Others	15.75	5.22	3.71	7.67	4.44	5.642
